# Trueness of full-arch dental models obtained by digital and conventional impression techniques: an in vivo study

**DOI:** 10.1038/s41598-022-26983-5

**Published:** 2022-12-29

**Authors:** Yonca Onbasi, Sabrin Abu-Hossin, Maria Paulig, Lara Berger, Manfred Wichmann, Ragai-Edward Matta

**Affiliations:** Department of Prosthodontics (Head: Prof. Dr. Manfred Wichmann), University Hospital, Friedrich-Alexander-University of Erlangen-Nuremberg (FAU), Glueckstrasse 11, 91054 Erlangen, Bavaria Germany

**Keywords:** Medical research, Optics and photonics

## Abstract

The aim of this study was to compare the trueness of complete- and partial-arch impressions obtained using conventional impression materials and intraoral scanners in vivo. Full-arch impressions were taken using polyether and polyvinylsiloxane. Gypsum casts were digitized using a laboratory scanner (IM, AF). Casts obtained from polyether impressions were also scanned using an industrial blue light scanner to construct 3D reference models. Intraoral scanning was performed using CEREC Omnicam (CO) and Trios 3 (TR). Surface matching software (Atos Professional) enabled to determine the mean deviations (mean distances) from the reference casts. Statistically significant discrepancies were calculated using the Wilcoxon signed-rank test. The mean distance for trueness ranged from 0.005 mm (TR) to 0.023 mm (IM) for the full arch, from 0.001 mm (CO) to 0.068 mm (IM) for the anterior segment, and from 0.019 mm (AF) to 0.042 mm (IM) for the posterior segment. Comparing the anterior vs. the posterior segment, significantly less deviations were observed for anterior with CO (*p* < 0.001) and TR (*p* < 0.001). Full-arch comparisons revealed significant differences between AF vs. IM (*p* = 0.014), IM vs. CO (*p* = 0.002), and IM vs. TR (*p* = 0.001). Full-arch trueness was comparable when using Affinis and the two intraoral scanners CEREC Omnicam and Trios 3. The digital impression devices yielded higher local deviations within the complete arch. Digital impressions of the complete arch are a suitable and reliable alternative to conventional impressions. However, they should be used with caution in the posterior region.

**Trial registration: **Registration number at the German Clinical Trial Register (04.02.2022): DRKS00027988 (https://trialsearch.who.int/).

## Introduction

There exist several available intraoral scanning systems that use advanced technology. The scanning of an impression or gypsum model is being progressively replaced by direct powder-free intraoral three-dimensional data collection^1–4^. The adoption of such techniques over conventional impression methods is mainly due to the reduced overall treatment time, ease of use, higher inter-operator reproducibility, and the ability to perform a quick repeat if imprecise scanned areas are noticed^2,5–12^. When fixed dental prostheses are fully digitally fabricated using computer-aided design and computer-aided manufacturing (CAD-CAM), without any gypsum cast, they often provide a better marginal and internal fit^13,14^.

The digital workflow is already routinely used in dental surgery, especially in implantology, and is increasingly utilized for the design and production of dental splints, and fixed dental protheses. Digital workflow use is now well-established for single crowns and fixed partial dentures with up to six units^6,11,13,15–20^. There are numerous indirect approaches for comparing the conventional and digital workflows, including the examination of restoration fit after each working technique^13,14,16,21,22^. Best-fit alignment is the most common direct method for accuracy evaluations of different impressions^7,12,23,24^.

In general, the scanned field can include individual teeth up to segments, quadrants, or even the full arch. Based on the present knowledge, the scanning of small divisions yields digital impressions with clinically satisfactory accuracy. However, the scanning of larger fields presents a challenge, especially if they are of similar quality. Therefore, the use of digital methods must be improved for removable dentures, such as total dentures^25,26^.

Regarding the complete arch, researchers have mainly focused on in vitro studies, in which they use a typodont or other individual model as a reference model and record the mean deviation therefrom^12,24,27–30^. Current studies indicate that digital imaging of the complete jaw is still a challenge^12,31–33^. There is a scarcity of investigations involving in vivo full-arch comparisons, and thus there remains a high demand for clinical studies of this matter^12,28,34–36^.

The aim of the present study was to respond to this request by comparing the trueness of complete-arch impressions obtained using two conventional impression materials and two intraoral scanners in vivo. It should be clarified whether intraoral scanning devices can replace full-arch conventional impressions under clinical conditions. The null hypothesis was that there are no statistically significant differences in trueness between the conventional and digital impression techniques.

## Materials and methods

### Participants and inclusion criteria

For this study, 31 adult participants (23 females, 7 males; mean age: 24 years, range: 22–32 years) with complete natural dentition (at least from the second molar to the contralateral second molar), good oral hygiene, and no ongoing dental or orthodontic treatments were recruited. Exclusion criteria were teeth damaged by caries or periodontal disease, present dental prosthesis, severe crowding, dentofacial deformity, or any intolerance or allergy to the utilized materials. All participants had to give their written consent. The experimental procedures were approved by the Medical Ethics Committee of Friedrich-Alexander University Erlangen-Nuremberg (approval number: 416_18B), and the study was registered in the German Register for Clinical Studies (reference number: DRKS00027988).

### Conventional and digital impressions

For all participants, full-arch conventional impressions of the upper jaw were taken both with a polyether impression material (Impregum Penta Soft; 3M ESPE, Neuss, Germany) as a monophasic impression, and with an addition curing silicone material (Affinis heavy/regular body; Coltène Whaledent, Langenau, Germany). Since the two-step impression was described to be more accurate compared to the one-step technique the two-step two-viscosity technique was used^37,38^. Thus, to obtain the silicone impressions, the tray was first filled with the heavy body, using a pentamix device. After removal from the mouth, the cured heavy body was prepared to be corrected by cutting it out and using a carving knife to create uniform ventilation channels on each tooth of the subject for an even layer thickness of the regular body. Then a second impression was taken with the regular body above the heavy body. To prevent dental movements, the conventional impressions were taken on two different days. To ensure a consistent impression technique and seating pressure, all impressions were taken by only one operator who had years of experience, and ventilation channels were created as already mentioned. Consistent ambient humidity and room temperature were maintained while taking conventional impressions, and all manufacturers’ instructions were followed. Materials were cleaned with an immersion disinfection (Eurosept Plus Impression Disinfection Liquid; Henry Schein, Langen, Germany) and subsequent rinced with water. The conventional impressions with Impregum Penta Soft and Affinis were air-dried and poured after 24 h with type IV dental stone (Fujirock EP-Classic; GC Corporation, Bad Homburg, Germany). To obtain a highly accurate reference model for each participant, the gypsum casts of the polyether impressions were digitized using a high-resolution industrial 3D-scanner (Atos Professional; GOM, ZEISS company, Braunschweig, Germany) and standard tessellation language (STL) files were created. This three-layer scanning technology has an average measurement error of 3 μm^39^. All gypsum casts (Polyether and Affinis) were additionally digitized (IM, AF) using an extraoral laboratory scanner (D900; 3shape, Düsseldorf, Germany) to generate STL-files for analyses. Thus, the casts of the Impregum impressions were scanned twice to detect the reliability and possible error sources of the employed laboratory scanner.

**Figure 1:**
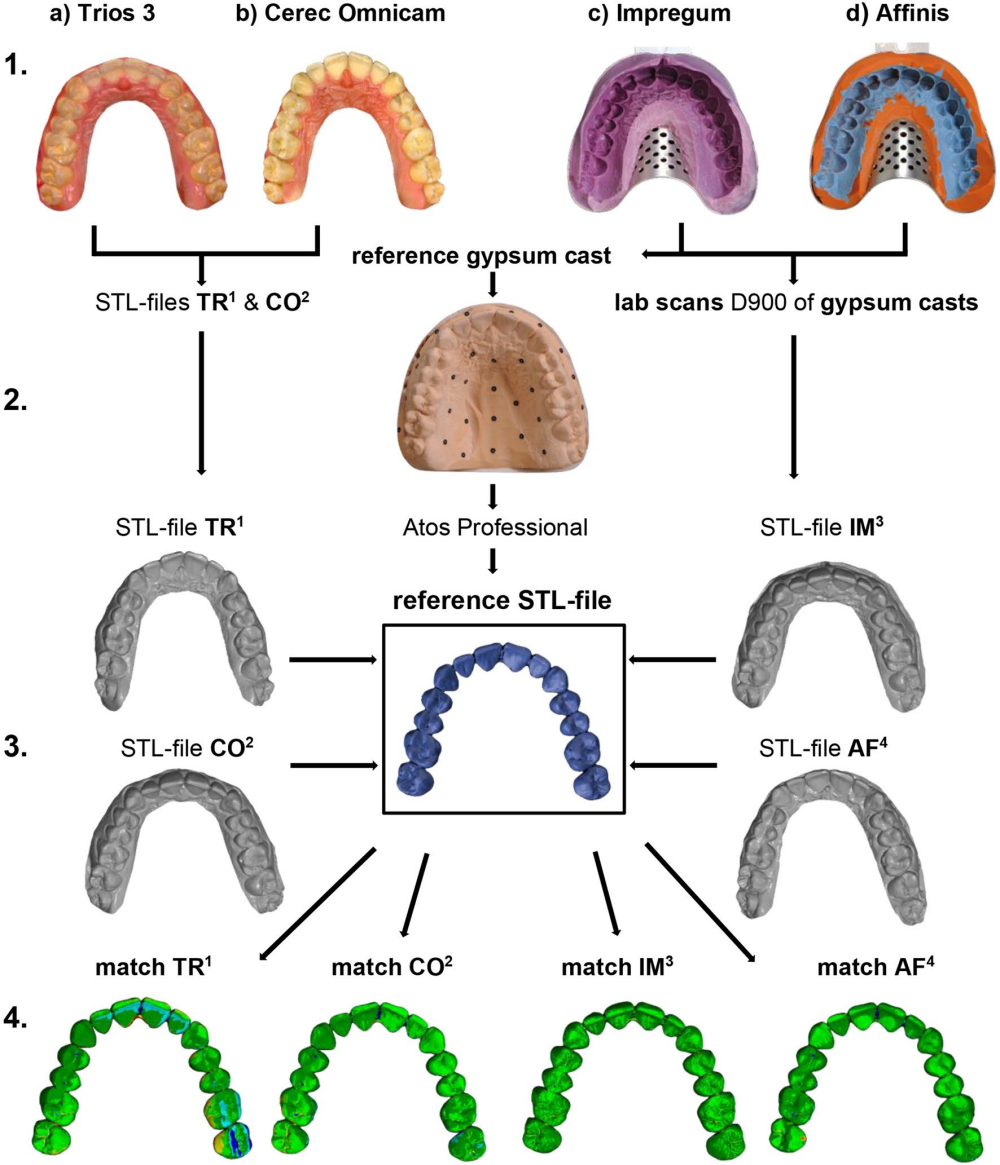
Flowchart illustrating the experimental design of the study based on an example of a test person. In the first step, the digital (**1a**) TRIOS 3 [TR^1^], (**b**) Cerec Omnicam [CO^2^]) and conventional, (**1c**) Impregum [IM^3^], (**d**) Affinis [AF^4^]) impressions were taken. Then, the reference gypsum cast of the IM-impression was provided with reference points and scanned with ATOS (GOM, ZEISS company) (**2**). Subsequently, the STL files of the different impression techniques were virtually superimposed on the STL reference model (**3**) so that the deviations were represented in the form of color-coded distance maps (**4**).

Direct digital full-arch models were obtained using two powder-free intraoral scanners: CEREC Omnicam v. 5.0.2 (Dentsply Sirona; Bensheim, Germany) and Trios 3 v. 1.6.10.1 (3shape; Düsseldorf, Germany). The manufacturers’ scanning strategies were also followed, which can be found in the user manuals of each system. The operator had previous training with the utilized scanners. For the comparisons, the data from the digital models were converted into standard tessellation language files (STL) (Fig. 1).

### Analysis

All data were stored in STL files for analyses. For three-dimensional difference analysis, surface matching software (Atos Professional; GOM, ZEISS company, Braunschweig, Germany) was used to superimpose the scans according to the best-fit-alignment method. The mean deviation values (mean distance in mm) were obtained by superimposing the respective model for each participant with the associated reference model. This method allows determination of the trueness of different impression methods.

First, the reference model was imported as an STL file, and subsequently trimmed to the tooth shape. The file for comparison was also imported as an STL file. Then, a rough, manual 3-point alignment of the two relevant models had to be implemented via prominent points on the tooth surfaces of the canines and molars for all study participants. Subsequently, the precise adjustment in form of a best-fit alignment could be performed by manual selection of the entire tooth surfaces, enabling the surface matching software to superimpose the models for variance analyses. Surface discrepancies between the two images were represented using color-mapping methods, and numbers. The color maps show a tolerance range of ± 10 µm (green) and a specified range of ± 100 µm (20 color segments). The analysis included all tooth surfaces. The complete arch was considered for evaluation, and a division into anterior and posterior segments was also realised. The total arch included teeth 17 to 27, the anterior segment teeth 13 to 23, and the posterior segment teeth 14 to 17, and 24 to 27. Thereby, the two segments were not scanned separately but were selected from the full-arch dataset of the digitized reference casts.

A statistician performed the evaluation. The level of significance was set at α = 0.05. Statistically significant discrepancies were calculated using the Wilcoxon signed-rank test. Statistical analysis was performed using the statistical software R V3.6.3, R Core Team 2020.

### Ethical approval and informed consent

The experimental procedures were approved by the Medical Ethics Committee of Friedrich-Alexander University Erlangen-Nuremberg (approval number: 416_18B). Before starting the data acquisition, all participants signed an informed consent. Before starting the data acquisition, all participants signed an informed consent. All persons gave their informed consent prior to their inclusion in the study. Details that might disclose the identity of the subjects under study were omitted. All methods were carried out in accordance with relevant guidelines and regulations in the declaration section.

## Results

**Figure 2:**
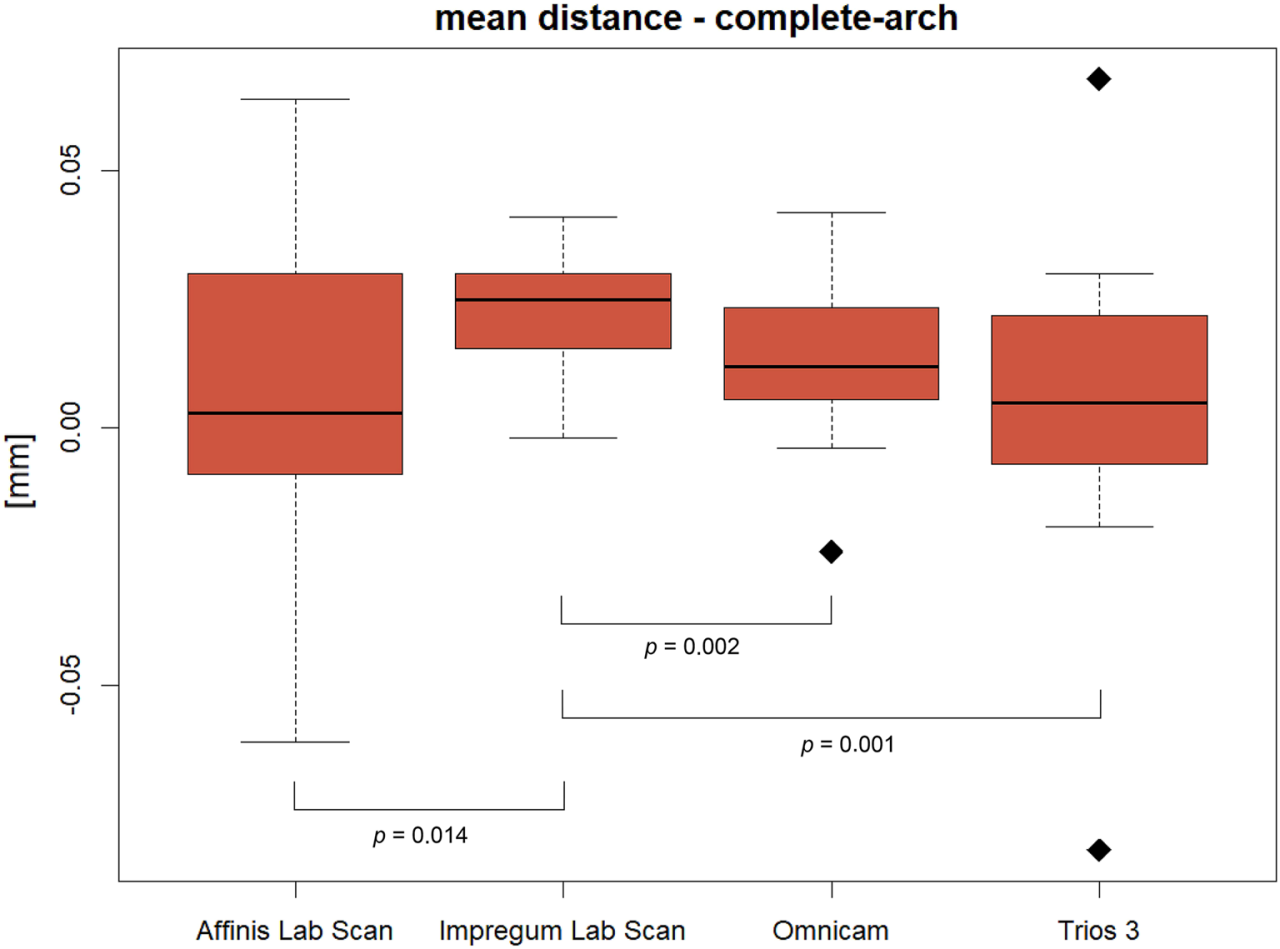
Mean distance for complete-arch (n = 31) and significant *p*-values.

**Figure 3:**
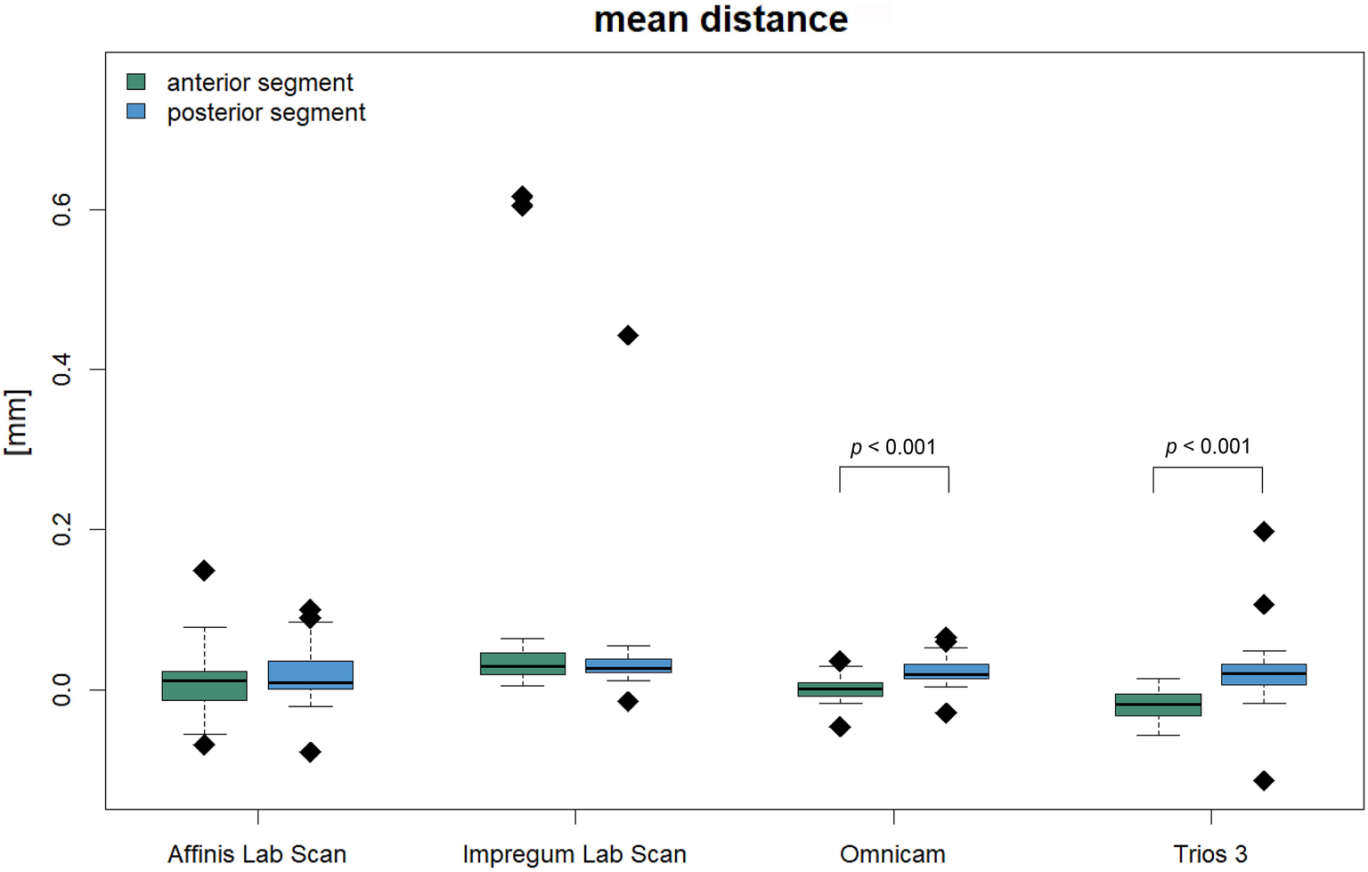
Mean distance for the anterior and posterior segment of dental arch (n = 31) and significant *p*-values.

The mean distance values (± standard deviation) for the complete arch ranged from 0.005 mm (± 0.025 mm) for Trios 3 (TR) to 0.023 mm (± 0.010 mm) for lab scans of the gypsum casts obtained using Impregum (IM). The mean values for the anterior segment ranged from 0.001 mm (± 0.016 mm) for CEREC Omnicam (CO) to 0.068 mm (± 0.146 mm) for IM, and those for the posterior segment ranged from 0.019 mm (± 0.036 mm) for lab scans of the gypsum casts obtained using Affinis (AF) to 0.042 mm (± 0.076 mm) for IM (Table 1). The mean distances from the reference models were generally similar between the conventional impression method using the silicone material AF and the two intraoral digital scanners. The impressions made using the polyether material IM exhibited the highest discrepancies for all compared areas (Table 1; Fig. 2, 3).

**Table 1:** Mean distance and standard deviation values of all impression groups: for complete arch, anterior and posterior segment in mm (n = 31).

Impression group	Relevant area of dental arch					
	Complete arch		Posterior segment		Anterior segment	
	Mean	SD	Mean	SD	Mean	SD
AF	**0.009**	0.028	0.019	0.036	0.010	0.039
IM	0.023	0.010	0.042	0.076	0.068	0.146
CO	0.014	0.014	0.023	0.019	0.001	0.016
TR	0.005	0.025	0.022	0.048	0.018	0.020

*AF* Affinis lab scan, *IM* Impregum lab scan, *CO* CEREC Omnicam, *TR* Trios 3, *Mean* mean distance, *SD* standard deviation.

Full-arch comparisons between all impression methods (AF vs. IM, AF vs. CO, AF vs. TR, IM vs. CO, IM vs. TR, and CO vs. TR) revealed significant differences between AF vs. IM (*p* = 0.014), IM vs. CO (*p* = 0.002), and IM vs. TR (*p* = 0.001) (Fig. 2). Specifically, the lab scans of gypsum casts obtained using the polyether impression material (IM) were significantly less precise compared to the silicone impression material (AF) or to both utilized intraoral scanners (CO and TR).

**Figure 4:**
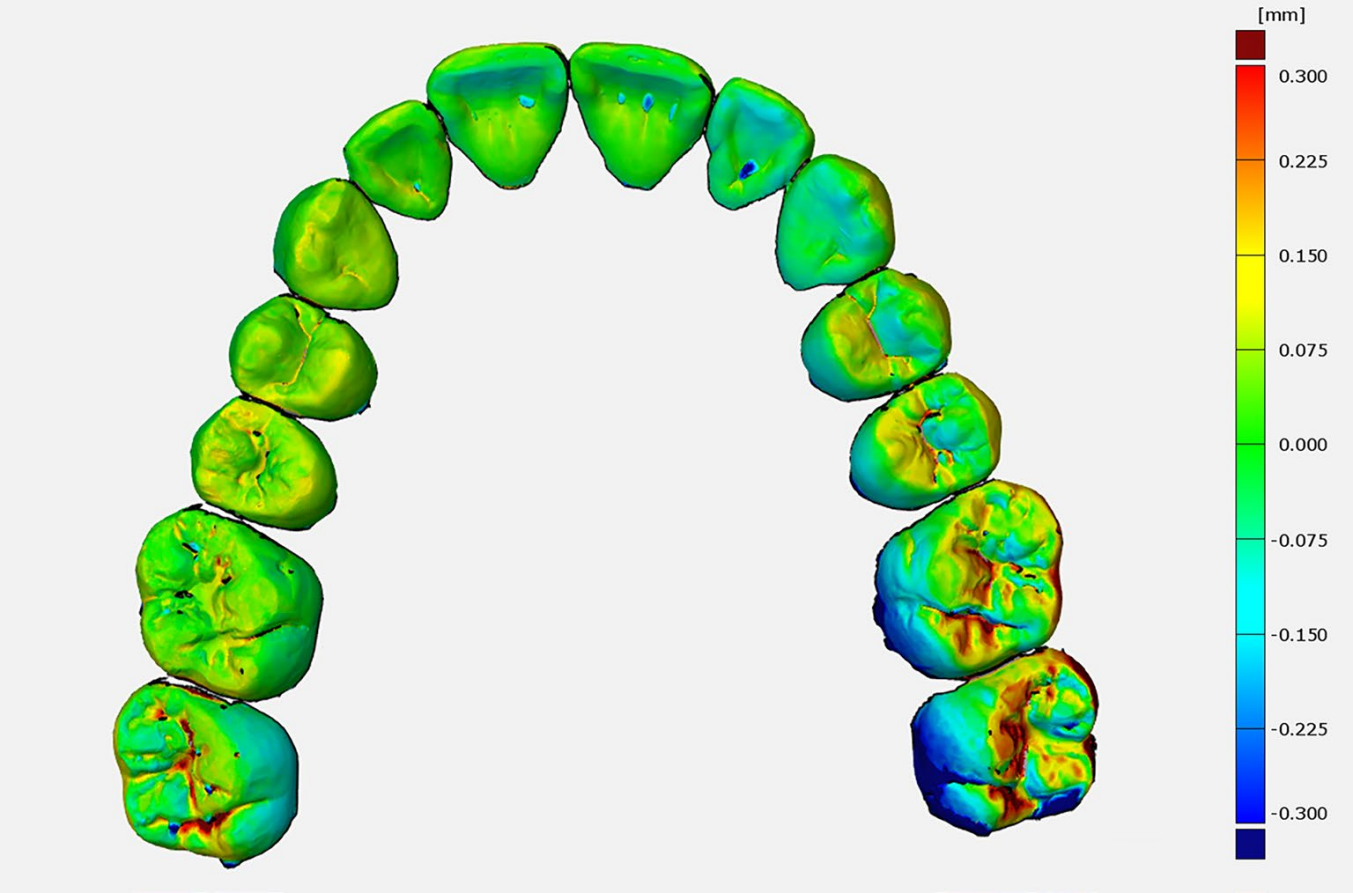
Color coded, 3D superimposition showing deviations of the Trios 3 scan to the reference cast between the anterior and posterior segments based on the example of one proband.

Comparative analysis between the anterior and posterior segment of the dental arch within the same impression group revealed significantly higher values for the posterior segment when using the intraoral digital scanner Omnicam (*p* < 0.001) or Trios 3 (*p* < 0.001) (Figs. 3, 4). Thus, with both scanners, the scans of the anterior segment showed significantly less deviations compared to the scans of the posterior segment. The conventional impressions were continuously accurate, with no significant differences between the anterior and posterior areas (*p* > 0.05).

## Discussion

In the present study, the trueness of conventional and digital impression methods for the complete arch was evaluated in vivo. In 31 participants, impressions of the upper jaw were taken with two routinely used conventional impression materials—a polyether and a vinylpolysiloxane material—and two intraoral scanners.

The two most routinely materials for high precision impressions were used for conventional impressions^40–43^. Polyether even gave more accurate impressions than polyvinylsiloxane. It is assumed that this is due to the hydrophilic nature of polyether and its use in the moist intraoral environment^44–46^. Assuming that polyether is the most accurate conventional impression material, Impregum Penta Soft was selected for obtaining the reference models. Clinical experience, and previous investigations by other authors, indicate that polyether is a highly accurate material^46–48^. Comparison studies have usually used the gypsum cast of a conventional impression as the reference model^12,29,36^. Similar to the study design of Ender et al., the anterior and posterior segments were considered separately for analysis after being digitally cut out of the complete arch^12^. The intention was to test in vivo whether there are differences in the detection of the dental arch between the anterior and posterior areas due to certain influencing factors. The posterior area was previously described as most prone to deviation in the case of intraoral scanning^49–52^. The method of best-fit alignment was used, as this is the most common direct concept for accuracy evaluations^12,23,24^.

The present results disproved the null hypothesis that there were no statistically significant differences between the conventional and the digital impression techniques for full-arch and partial-arch comparisons.

The accuracy of the impression indisputably determines the fit of the produced prosthetics. There is still no clear evidence how much discrepancy of a dental prosthesis is clinically acceptable. However, many authors propose a clinically acceptable marginal gap of up to 120 µm (incl. 25–50 µm for a cement layer)^53–55^. In the present study highly satisfactory mean distance values from 1 µm to 68 µm were obtained (full arch: 5 µm with TR to 23 µm with IM, anterior segment: 1 µm with CO to 68 µm with IM, and posterior segment: 19 µm with AF to 42 µm with IM)^12,29^. The in vitro investigation of Ender et al. showed similar mean deviation values of 16 µm to 93 µm for the complete arch (polyvinylsiloxane vs. Cerec Omnicam v. 4.6.1), 14 µm to 69 µm for the anterior part, and 10 µm to 47 µm for the posterior part (both polyvinylsiloxane vs. Medit i500 v. 1.2.1). They also compared the maxilla, in which they applied various intraoral scanners, and one conventional impression material. The impression with polyvinylsiloxane showed a significant better match with the custom complete-arch reference cast^12^. In contrast, in the present study, the values obtained with AF were generally very similar to those obtained with the digital scanners. Other investigations have reported similar results for conventional impression material and digital devices^30,56^. Especially regarding the complete arch, authors have mainly focused on in vitro studies using representative dental models. Ender et al. concluded that digital impressions could compete with conventional impressions in cases of partial arch capturing^12,30^. However, they and other authors have reported that conventional impression methods still showed higher accuracy when capturing the complete arch^12,27,29,57,58^. This may be largely because digital intraoral scanners only record sections of the dental arch and then match them to a whole image. The software identifies overlapping areas and superimposes them electronically, which may result in inaccuracies^12,28,59^. Nevertheless, some authors have reported that certain devices can keep up with conventional methods for the full arch^30,34,35,60^.

Concerning the fabrication of dental prosthesis, many authors have described intraoral scanning devices as being more precise than conventional impressions when producing single crowns or short-span restorations^6,16,18,21–23,36,58,61^. However, limited data are available regarding the survival of these restorations. Therefore, a meta-analysis concluded that these results should be interpreted with caution^62^. On the other hand, conventional methods are reported to be more precise when producing large or full-arch restorations^58^. To date, there is no general agreement on which impression method is most suitable.

The significance analysis revealed that the laboratory scans of gypsum casts made with Impregum (IM) significantly differed from the dental models obtained from impressions made with Affinis (AF) or from the two digital intraoral scanners, CEREC Omnicam (CO) and Trios 3 (TR). Specifically, IM showed significantly less trueness in full-arch comparisons when superimposed with the corresponding digitized reference casts. Better values for IM were expected, since prior studies showed similar accuracy from a polyether and a silicone impression material^63^. However, it can be assumed that the method used to make the reference casts was reliable as the polyether impressions were poured and directly scanned using the high-resolution industrial 3D-scanner Atos Professional. These casts, together with the gypsum casts of the silicone impressions, were digitized using an extraoral laboratory scanner. This could lead to errors, since the casts obtained using the impressions with polyether were transported and scanned twice. It appears that the indirect digitization of stone casts is fraught with errors and may provide inaccurate results^14,23,34,64^. Moreover, by scanning the polyether plaster model with both the high-resolution reference scanner and the extraoral laboratory scanner, the measurement accuracy of the latter scanner should be checked. The superimposition of the two digital models obtained should show maximum match for superimposition. Regarding Fig. 3 statistical outliers in case of IM can be seen at once. Thus, this is due to a measurement inaccuracy of the lab scanner.

The two utilized intraoral scanners showed significant differences when comparing the anterior segment versus posterior segment within the full arch of the same impression group. Superimposition of the anterior segments of the digital intraoral scans with the same fields of the relevant reference models indicated significantly higher trueness compared to the posterior segments. This can be explained by the more difficult conditions for scanning in the posterior area of the jaw, due to the limited oral space for the scan head and the higher condensation from breath or saliva. This finding is in accordance with the observations of Zhang et al., Ender et al., and Patzelt et al., who all found that higher local deviations within the full arch are possible when using a digital intraoral scanner^35,36,57,65^.

Only few clinical studies have compared digital versus conventional impressions of the complete arch. A thorough search revealed only four such studies. Zhang et al. had the largest number of participants (n = 20), and performed the only study approaching the number of participants in the present study^36^. Zhang et al. and Sfondrini et al. only used the conventional impression material Alginate for comparisons, and each only used one digital intraoral scanner^36,64^. Zhang et al. found no significant differences between the gypsum casts and the digital models generated by the scanner iTero, and concluded that digital impression methods are clinically acceptable, and could replace dental stones^36^. Sfondrini et al. examined 14 participants and determined that the gyspsum casts were not significantly different from the scans using Trios 3. Their conclusions emphasized that intraoral scanning seems to be more time efficient and more comfortable for orthodontic applications, and is on track to replace conventional methods^64^. However, it must be noted that both of these studies used Alginate, which is not a highly precise conventional material^36,64^. Moreover, the poured gypsum casts were not scanned using a high-precision industrial scanner to obtain the reference models, but rather were scanned using the same scanner that was employed for the intraoral scans^64^. Ender et al. applied various conventional materials, including polyether, vinylsiloxanether, and Alginate, and employed seven digital scanners on a total of five participants. They found higher precision when using conventional impressions, except for the alginate group. In contrast to the two previously mentioned studies, they found that the scans were more accurate than the alginate impressions^57^. This result was more likely to be expected. Keul and Guth examined the upper jaw of only one participant, using an addition curing silicone as a conventional impression material, and using iTero Element for intraoral scans. A unique aspect of their study was the implementation of an in vivo and an in vitro comparison. For the in vitro part, the silicone impression of the patient was casted with a model resin. Their results from both parts of the study indicated that iTero yielded the same or even higher accuracy for single parameters^34^. Other clinical investigations have focused on single teeth up to segments of the dental arch^7,56,66^.

The limitations of the present study were that no repetitions were conducted. This was due to the in vivo nature of this study, the relatively large number of participants and their compliance, and the associated exposure time. Similarly, impressions are not repeated in the clinical routine if all therapeutically relevant structures have been represented. Thus, in line with the everyday practice, only trueness and not precision was investigated. Some authors have likewise used only one component to describe accuracy^49,67–70^. Moreover, to enable an in vivo comparison, an impression method had to be selected for processing the reference models. This was previously established^56^. Therefore, no standardized independent model could be applied, as could be done in an in vitro study. Additionally, the anterior and posterior segments were not scanned separately but were rather generated from the data of the complete-arch scan, as was previously described in another study^12^.

## Conclusion

Within the limitations of this in vivo study, scanning the complete dental arch with the tested digital impression systems yielded clinically acceptable results, and represents a suitable and reliable alternative to conventional impressions. The trueness was similar between Affinis and the two intraoral scanners, CEREC Omnicam and Trios 3. Digital impression devices can show higher local deviations within the complete arch. More precise results were obtained for the anterior segment compared to the posterior area. Depending on the clinical problem, the conventional impression can be replaced by the digital impression. Taking into consideration that digital devices should be used with caution in the posterior region.

These results should be verified in additional clinical studies, which should preferably include both jaws and more digital intraoral scanners.

## Data Availability

The datasets generated and/or analysed during the current study are not publicly available due part of this data from the study will also be used in the upcoming dissertation by Maria Paulig, and it includes patient data but are available from the corresponding author on reasonable request.
